# Supplementary material for: Impact of the Covid-19 pandemic on inpatient health care in Switzerland 2020–2021—A descriptive retrospective study using admission data of all Swiss hospitals
Source: PLoS One. 2024 Jul 8;19(7):e0306791. doi: 10.1371/journal.pone.0306791 (PMC11230527; doi:10.1371/journal.pone.0306791)
Supplement: Attachment — Submitted filename: Response to reviewers.docx [file pone.0306791.s001.docx]

**Response to reviewers**

Dear reviewers

Thank you for your detailed review and valuable suggestions for enhancing our manuscript. We have addressed your comments in the blue-text sections below and have outlined the corresponding revisions. Line numbers refer to the manuscript with tracked changes.

Reviewer #1: Comments: In this study, the authors described the changes of overall and procedure-specific hospital admissions in Switzerland during the COVID-19 pandemic in 2020 and 2021.

Main concern:
The authors estimated the changes in hospital admissions by directly comparing the differences between periods during the pandemic and corresponding periods in 2019. The problem with this method is that it couldn't account for the long-term trend of hospital admissions. Although the authors explained in the methods, the percentage changes in 2020 and 2021 were small (5.8% and 0.9% less than in 2019), there is the possibility that the reduction was attributed to the long-term reducing trend rather than the effects of the pandemic. I can see from Figure 1 that the authors had the data during 2016 and 2021. Therefore, I would like to suggest predicting the weekly number of hospitalizations in 2020 and 2021 using time-series data from 2016-2019 by fitting Poisson or Negative Binomial Distribution Models, accounting for the long-term trend and seasonality. Then to compare the difference between observed and predicted values in the study period to estimate the absolute or percentage changes. This method could also solve the uncertainties issue, which is a problem in this study. If the authors insist on using the current method (comparing study periods with corresponding periods in 2019), I would like to suggest another method used in the Filippo et al._ J Med_2020 paper, which would help to solve the uncertainties issue.

Thank you for this comment. We recognize the importance of selecting an appropriate comparator in our analysis. After extensive discussions on the methodological approach, we selected 2019 as the reference year without additional adjustments. In the manuscript, we argue why choosing the year 2019 as a comparator is, in our view, the best choice. Our decision favored a more straightforward approach over complex modeling that would assume a constant trend, linear or otherwise.

The reviewer raises the concern that the decrease in case numbers might be attributable to a long-term reducing trend rather than the effects of the pandemic. This is clearly not the case, as there is no decreasing trend in case numbers from 2016 to 2019 (minimal annual changes ranging from -0.12% to +0.24% compared to 5.77% for the year 2020).

Controlling for seasonality was a key consideration in our methodology. By analyzing weekly admission numbers, our study inherently accounts for seasonal variations in hospital admissions.

We refined and amended the corresponding part in the methods section as follows:

line 160ff: " We compared admission numbers from 2020 and 2021 with those from 2019. Because inpatient care **in a particular field** can be subject to considerable systematic changes from one year to another, the meaningfulness of the comparison decreases with each year further back in time. Because changes might occur abruptly, statistical modeling using linear and non-linear trends is limited and rests on questionable ceteris paribus assumptions. 2019, the year prior to the Covid-19 pandemic, thus corresponds to the best possible control period, since it can be assumed that disease prevalence, hospital infrastructure, medical coding guidelines and classification systems, reimbursement schemes, as well as other potentially relevant factors change little within a year. **Similarly, other trends, including population aging, likely had minimal impact on our findings, given the limited timeframe of our analysis. This is evidenced by the minimal yearly variation in total case numbers from 2016 to 2019, with annual changes ranging from -0.1% to +0.2%, compared to a 5.8% decrease in 2020.** "

We are grateful for the reviewer's suggestion to use the analysis method as in the Filippo et al._ J Med_2020 paper. While, as argued above, we do not see the benefit in modeling, we indeed see the point in accounting for uncertainty for analyzing the "selected procedures" where case numbers are comparatively low.

Therefore, we included incidence rate ratios (IRR) and their respective confidence intervals in table 2. This addition aims to offer a more precise understanding of our findings' statistical significance and variability. We continue to present percentage-point changes in the first place, facilitating an intuitive grasp of the shifts in case numbers.

In the methods section, we explain this procedure as follows:
line 177ff: "We report Incidence Rate Ratios (IRRs) and corresponding 95% confidence intervals derived from a univariable Poisson regression when analyzing selected groups of procedures, some of which had low case numbers. The IRRs compare incidence rates between two time points (2020 and 2021 vs. 2019), calculated as $IRR=\frac{inpatient stays_{2020/2021}}{inpatient stays_{2019}}$. An IRR of 1 indicates no difference in incidence rates between the two periods. We assume a constant population size and characteristics over the comparison period. The IRR is obtained by exponentiating the regression coefficient for the comparison year. For background on Poisson regression, see reference [32], and for a recent application to hospital admissions, see reference [33]. This method allows us to address estimation uncertainty, especially pertinent in our analysis of the selected procedures with low case numbers."

Minor concern
In figures 2 and 3, I would suggest labeling each part (for example, "A", "B", "C", "D"), and to show us the overall percentage changes for each group rather than the weekly changes.

We have updated figures 2 and 3 to include labels for each part as suggested (e.g., "A", "B", "C"), facilitating easier reference. Additionally, we have incorporated the total yearly percentage change for each group in these figures and refer to it in the text. We maintain the presentation of weekly changes as they provide essential insights into the timing and dynamics of the observed changes in hospital admissions across different periods of the pandemic. This level of detail is crucial for understanding the specific impacts of the pandemic on hospital admissions.

Reviewer #2: The article appears well written and has a good research methodology which has been clearly stated. The topic is certainly interesting and useful not only for observation purposes but also for research purposes and interest in the planning of health systems in the Western world, I therefore believe that the article should be published.
I have some suggestions:
- the authors examine the results of their analysis which appear globally comparable to other international data and try to give explanations regarding them. I have not seen that a fundamental aspect for the healthcare systems of European countries, namely the increase in the average age, is taken into consideration. The authors could mention this variable indicating whether this had any interaction in the study.

Thank you for acknowledging the strengths of our manuscript and the suggestion regarding the increase in average age. We have considered the impact of aging on the healthcare systems; however, given our study's focus on the short-term effects of the COVID-19 pandemic, the aging population trend did not significantly affect our results. We now explicitly state this in our manuscript to clarify the scope of our analysis:
Line 169ff: " Similarly, other trends, including population aging, likely had minimal impact on our findings given the limited timeframe of our analysis."

- the results show a decline in access to the emergency room/emergency during the lockdown period but also subsequently; the fact that following the lockdown but also in the subsequent period there were restrictions on the traffic of people or in any case there was less traffic on the road, this aspect may have reduced car accidents and therefore access for this reason. Could this be a reason to add and discuss briefly?

Thank you. Trauma cases did indeed decline. We have added a brief mention of this aspect to our discussion, referencing a study that examined trauma cases in Switzerland and in the US during the same timeframe. However, it's important to note that our analysis primarily focused on patient groups not directly affected by trauma-related admissions, such as those undergoing elective surgery, cancer surgery, and patients with conditions like stroke, myocardial infarction, and appendicitis.
Line 350ff: "Also, particularly in lockdown periods, decreased mobility and restricted work and leisure activities led to a reduction in trauma patient admissions. For Switzerland, a decrease of 40 to 55% was noted during the spring 2020 lockdown[38], a trend that aligns with observations from other countries, including the US[39]."

- the conclusions are a bit lacking. Could some useful considerations be made for designing the future of healthcare systems to make them more resilient?

Our study's main objective was to systematically document and analyze the changes in inpatient case numbers during the pandemic. Due to the variability in the reasons for these declines and the differences in the time to compensate across patient groups, we refrained from proposing specific enhancements for bolstering the resilience of the healthcare system. However, our analysis revealed no lasting impact on inpatient health service utilization, underscoring the Swiss healthcare system's ability to adapt to the pandemic's demands swiftly. This resilience, often scrutinized for its perceived excess resources and high costs, highlights the critical role of resource availability in the system's effective response to the crisis.

We added a corresponding argument in the conclusion:
line 453ff: " The findings show the Swiss healthcare system's ability to swiftly adapt to the pandemic's demands. This resilience, often scrutinized for its perceived excess resources and high costs, highlights the critical role of resource availability in the system's effective response to the crisis."

Reviewer #3: The article is well structured however it does not provide any innovative elements. Substantially, it is a descriptive analysis of three years 2019, 2020, 2021 of hospitalizations but provides only a modest scientific contribution. it is, in fact, logical that, during the lockdown there was a decline in hospitalizations with a subsequent surge, this is a fact already widely described in the literature. It would have been useful, however, to find out whether the reduction in hospitalizations has led, for example, to a delay in the diagnosis of some cancers. In summary, I do not believe that the scientific contribution of this manuscript is useful today.

We agree with Reviewer #3 that the decline in hospitalizations during the lockdown, followed by a subsequent surge, is a widely observed phenomenon and not unique to the Swiss Health System. However, we believe that our systematic and detailed analysis of this decline and precise quantification offer valuable insights into how a health system operates under pandemic conditions. The usefulness of this detailed analysis should be determined by future readers and audiences who seek to understand the health system's functionality and resilience during such periods.

6. PLOS authors have the option to publish the peer review history of their article ([what does this mean?](https://journals.plos.org/plosone/s/editorial-and-peer-review-process#loc-peer-review-history)). If published, this will include your full peer review and any attached files.


If you choose "no", your identity will remain anonymous but your review may still be made public.


**Do you want your identity to be public for this peer review?** For information about this choice, including consent withdrawal, please see our [Privacy Policy](https://www.plos.org/privacy-policy).

Reviewer #1: No

Reviewer #2: **Yes:** Matteo Bolcato

Reviewer #3: No

[NOTE: If reviewer comments were submitted as an attachment file, they will be attached to this email and accessible via the submission site. Please log into your account, locate the manuscript record, and check for the action link "View Attachments". If this link does not appear, there are no attachment files.]

While revising your submission, please upload your figure files to the Preflight Analysis and Conversion Engine (PACE) digital diagnostic tool, <https://pacev2.apexcovantage.com/>. PACE helps ensure that figures meet PLOS requirements. To use PACE, you must first register as a user. Registration is free. Then, login and navigate to the UPLOAD tab, where you will find detailed instructions on how to use the tool. If you encounter any issues or have any questions when using PACE, please email PLOS at [figures@plos.org](mailto:figures@plos.org). Please note that Supporting Information files do not need this step.
